# The F-box protein COI1 functions upstream of MYB305 to regulate primary carbohydrate metabolism in tobacco (*Nicotiana tabacum* L. cv. TN90)

**DOI:** 10.1093/jxb/eru084

**Published:** 2014-03-06

**Authors:** Wenjing Wang, Guanshan Liu, Haixia Niu, Michael P. Timko, Hongbo Zhang

**Affiliations:** ^1^College of Agronomy and Biotechnology, Southwest University, Chongqing 400716, PR China; ^2^Tobacco Research Institute, Chinese Academy of Agricultural Sciences, Qingdao 266101, PR China; ^3^Department of Biology, University of Virginia, Charlottesville, VA 22904, USA

**Keywords:** COI1, jasmonate, primary metabolism, starch, tobacco.

## Abstract

Jasmonate (JA) plays an important role in regulating plant male fertility and secondary metabolism, but its role in regulating primary metabolism remains unclear. The F-box protein CORONATINE INSENSITIVE 1 (COI1) is a critical component of the JA receptor, and mediates JA-signalling by targeting JASMONATE ZIM-domain (JAZ) proteins for proteasomal degradation in response to JA perception. Here, we found that RNA interference-mediated knockdown of *NtCOI1* in tobacco (*Nicotiana tabacum* L. cv. TN90) recapitulated many previously observed phenotypes in *coi1* mutants, including male sterility, JA insensitivity, and loss of floral anthocyanin production. It also affected starch metabolism in the pollen, anther wall, and floral nectary, leading to pollen abortion and loss of floral nectar. Transcript levels of genes encoding starch metabolism enzymes were significantly altered in the pollen, anther wall, and floral nectary of *NtCOI1*-silenced tobacco. Changes in leaf primary metabolism were also observed in the *NtCOI1*-silenced tobacco. The expression of *NtMYB305*, an orthologue of MYB305 previously identified as a flavonoid metabolic regulator in *Antirrhinum majus* flowers and as a floral-nectar regulator mediating starch synthesis in ornamental tobacco, was extremely downregulated in *NtCOI1*-silenced tobacco. These findings suggest that NtCOI1 functions upstream of NtMYB305 and plays a fundamental role in coordinating plant primary carbohydrate metabolism and correlative physiological processes.

## Summary

NtCOI1, a tobacco orthologue of the *Arabidopsis* jasmonate co-receptor COI1, was shown to regulate primary carbohydrate metabolism in both reproductive and vegetative organs of *Nicotiana tabacum* L. cv. TN90.

## Introduction

Plants produce two types of metabolites: primary metabolites and secondary metabolites. Primary metabolites are essential for plant growth and development, and serve as substance and energy sources for secondary metabolism. Secondary metabolites are not required for plant survival but do facilitate plant primary metabolism and play a role in plant defence (Plaxton and McManus, 2006; Aharoni and Galili, 2011).

Jasmonate (JA) is a phytohormone that plays an essential role in the regulation of plant fertility, secondary metabolism, and defensive responses (Li *et al.*, 2004; Lorenzo and Solano, 2005; Nagpal *et al.*, 2005; Wasternack, 2007; Fonseca *et al.*, 2009; Pauwels and Goossens, 2011). Perception of the bioactive JA derivative, jasmonoyl-l-isoleucine (JA-Ile), by the receptor complex comprised of the F-box protein CORONATINE INSENSITIVE1 (COI1), JASMONATE ZIM-domain (JAZ) protein, and an inositol pentakisphosphate molecule, results in degradation of specific JAZ proteins by the 26S proteasome (Chini *et al.*, 2007, 2009; Thines *et al.*, 2007; Katsir *et al.*, 2008; Yan *et al.*, 2009; Sheard *et al.*, 2010). Subsequently, JAZ-interacting transcription factors, such as basic helix–loop–helixs (bHLHs), MYBs, and EIN/EIL, are liberated to activate JA-mediated responses (Adams and Turner, 2010; Cheng *et al.*, 2011; Fernandez-Calvo *et al.*, 2011; Pauwels and Goossens, 2011; Qi *et al.*, 2011; Song *et al.*, 2011; Zhu *et al.*, 2011). Thus, the F-box protein COI1 plays an indispensable role in the JA-signalling pathway in plants.

Male sterility, a dominant feature of plants with defective COI1 (Feys *et al.*, 1994; Xie *et al.*, 1998; Li *et al.*, 2004; Paschold *et al.*, 2007), is regulated by the COI1–JAZ complex via the action of MYBs in the JA-signalling pathway (Chung and Howe, 2009; Song *et al.*, 2011). In *Arabidopsis*, mutation of COI1 leads to loss of anthocyanin pigments (Feys *et al.*, 1994; Xie *et al.*, 1998; Qi *et al.*, 2011), whose synthesis is mediated by the JAZ-interacting WD-Repeat/bHLH/MYB complex downstream of the JA-signalling pathway (Gonzalez *et al.*, 2008; Qi *et al.*, 2011). Studies suggest that the COI1–JAZ complex plays a central role in regulating biosynthesis of alkaloids, a group of important secondary metabolites for plant defence, via the JAZ-interacting bHLH transcription factor MYC2 (Shoji *et al.*, 2008, 2010; De Boer *et al.*, 2011; Zhang *et al.*, 2012). These pieces of evidence indicate that the COI1–JAZ complex acts as the core JA-signalling module in male fertility and secondary metabolism.

Involvement of the JA-signalling pathway in primary metabolism has been implied (Jost *et al.*, 2005; Schwachtje and Baldwin, 2008). JA-signalling components play an important role in regulating pollen development and secondary metabolism (Feys *et al.*, 1994; Xie *et al.*, 1998; Chini *et al.*, 2009; Chung and Howe, 2009; Pauwels and Goossens, 2011; Qi *et al.*, 2011; Song *et al.*, 2011), and these physiological processes are highly correlated with primary metabolism in plants (van der Fits and Memelink, 2000; Horner *et al.*, 2007; Nashilevitz *et al.*, 2009; Zhang *et al.*, 2010; Xiong *et al.*, 2013). Recently, JA was implicated as a regulator of the production of floral and/or extrafloral nectar, a sugar-rich fluid secreted by plant nectaries (Heil *et al.*, 2001; Gonzalez-Teuber and Heil, 2009; Nepi *et al.*, 2009; Radhika *et al.*, 2010a, b; Heil, 2011). The MYB transcription factor MYB305, which acts as a floral-nectar regulator mediating starch synthesis in ornamental tobacco, is an orthologue of the *Arabidopsis* male fertility regulators MYB21 and MYB24 (Song *et al.*, 2011; Liu and Thornburg, 2012). However, the molecular mechanism by which JA controls the production of primary metabolites and energy sources needed to drive plant development and secondary metabolism remains unclear.

COI1 plays a comprehensive role in controlling overall JA signalling (Feys *et al.*, 1994; Sheard *et al.*, 2010; Pauwels and Goossens, 2011; Qi *et al.*, 2011; Kazan and Manners, 2012). Hence, determining the roles of COI1 in primary metabolism is critical for fully defining the underlying molecular regulatory mechanism by which the JA-signalling pathway regulates primary metabolism. To do this, we generated *NtCOI1*-silenced transgenic tobacco (*Nicotiana tabacum* L. cv. TN90), as tobacco has large reproductive organs and allows examination of the effects of *NtCOI1* silencing on various aspects of plant primary metabolism. The transgenic plants exhibited alterations in starch metabolism, and in the transcription profiles of *NtMYB305* and starch metabolic genes. Our study provides compelling molecular evidence that NtCOI1 functions upstream of NtMYB305 to control primary carbohydrate metabolism and male fertility.

## Materials and methods

### Plant materials, growth conditions, and transgenic plant production

Tobacco (*N. tabacum* L.) cultivar TN90 was used as a control and for the generation of transgenic plants. All tobacco plants were grown at 23 °C in a greenhouse with a photoperiod of 14h light/10h dark. Plant tissues were collected at different floral developmental stages (as described below).

Transgenic plants were developed using *Agrobacterium*-mediated transformation with *Agrobacterium tumefaciens* LBA4404 carrying either the *NtCOI1*-silencing vector or a vector control (VC). To construct the *NtCOI1*-silencing vector, forward and reverse primers (NtCOI1 forward primer 5′-CACCGATCTACCACTTGATAATGGTGTC-3′; NtCOI1 reverse primer 5′-TTCACTAAAACAGCAGCCTCTCAC-3′) were designed to amplify a *NtCOI1* fragment corresponding to a C-terminal region (aa 421–502; ~350 residues downstream of the F-box) of *Arabidopsis* COI1 (Supplementary Fig. S1 available at *JXB* online). This fragment was amplified by reverse transcription (RT)-PCR from tobacco cv. TN90 total RNA, and inserted into the pENTR-D-TOPO® vector (Invitrogen). Following sequence confirmation, the fragment was mobilized to the 2× cauliflower mosaic virus (CaMV) 35S promoter-containing Gateway® compatible RNA interference (RNAi) vector pHZPRi-Hyg (Zhang *et al.*, 2012) by Gateway LR recombination with Gateway^®^ LR Clonase™ (Invitrogen) according to the manufacturer’s instructions. The VC was constructed by a Gateway LR recombination reaction with the empty pENTR-D-TOPO® vector and the RNAi vector pHZPRi-Hyg. Both wild-type and VC-transformed plants were used as control plants.

The *NtCOI1*-silenced plants were male sterile. Therefore, their seeds were produced by pollination using pollen grains from wild-type plants. Antibiotic (hygromycin)-resistant seedlings were selected to produce seeds for each generation. Seven-day-old hygromycin-resistant T_1_ seedlings were used for the JA-sensitivity assay (Supplementary Fig. S3 available at *JXB* online).

### Characterization of floral development stages

In this study, the floral development stages 2–12 (S2–S12) of tobacco were in accordance with those defined by Koltunow *et al.* (1990) and Drews *et al.* (1992). The floral development stages 13–16 (S13–S16; Supplementary Fig. S2 available at *JXB* online) and characterization of the ovary, floral nectary, and floral nectar at relevant floral development stages are defined in this work. The characteristics of these floral development stages are as follows: S2: pale green corolla emerged from dark green calyx, microspores separated; S3: corolla above sepal tips, tapetum shrunken, pollen grains begin to form; S4: sepals completely separated at top of calyx, corolla above sepal tips, tapetum degenerating; S6: corolla tube bulge at tip of calyx, remnants of tapetum present; S8: corolla elongating, petals green and slightly open, ovary pale green, floral nectary turning orange; S9: corolla tube bulge enlarging, petal tips becoming pink, ovary milky-yellow, floral nectary pale orange; S10: corolla limb beginning to open, petal tips pink, ovary milky-yellow, floral nectary orange; S11: corolla limb halfway open, ovary milky-yellow, floral nectary bright orange, floral nectar visible; S12: flower open, corolla limb fully expanded and deep pink, tube white in appearance, pollen released, ovary milky-yellow, floral nectary bright orange, large amount of floral nectar; S13: flower open, corolla limb pale purple, tiny amount of floral nectar, ovary visibly filled, visible chlorophyll accumulation, floral nectary milky-yellow and shrinking; S14: corolla limb deep purple and wilting, floral nectar invisible, ovary green and nearly half-filled; S15: corolla limb dark purple and wilted, tube pale grey, ovary deep green and over half-filled; and S16: corolla and pistil dried off, tube pale brown, ovary deep green and almost fully filled.

### Transient expression and detection of NtCOI1 protein

To test the specific disruption of NtCOI1 protein by RNAi-mediated gene silencing, agroinfiltration-mediated transient expression of a haemagglutinin (HA)-tagged NtCOI1 fragment in the tobacco leaf was conducted, and protein expression was tested by western blotting. To construct the vector expressing HA-tagged NtCOI1, the triple HA-tag-coding segment was fused in frame into the 3′ end of the fragment encoding the NtCOI1 region corresponding to aa 285–502 of AtCOI1 by PCR amplification. This fusion fragment was inserted between the *Swa*I and *Xba*I sites of the binary vector pBin19–attR–YFP (Subramanian *et al.*, 2006) to generate the pBin19–NtCOI1–HA vector. Agroinfiltration and western blot assays were performed as described by Voinnet *et al.* (2003). The infiltrated area was marked immediately after infiltration, and then cut off from plants after 4 d of growth in the greenhouse for protein extraction and western blotting with an antibody against the HA tag (BBI Company).

### Pollen germination assay

Pollen grains from just-dehisced anthers were directly released onto plates containing modified pollen germination medium [15% (w/v) sucrose, 0.01% (w/v) boric acid, 5mM Ca(NO_3_)_2_, 1mM MgSO_4_, 5mM CaCl_2_, pH 7.5, with 1.5% (w/v) agar; Arun *et al.*, 2011], and germinated for 10h at 23 °C in darkness. The pollen was observed and photographed under a microscope.

### Carotenoid analyses

To extract nectary carotenoids, 20 nectaries at the indicated stages were separated from their ovaries, ground into powder with liquid nitrogen, and extracted with 3ml of acetone in a centrifuge tube. After 10min of extraction at room temperature, samples were centrifuged at 3500*g* for 10min. Supernatant extracts were then dehydrated with anhydrous sodium sulfate.

For the thin layer chromatography (TLC) assay, equal volumes of the extracts were loaded onto a TLC plate, and separated with modified petroleum ether:isopropanol:water (100:10:0.1) solvent (Pocock *et al.*, 2004).

Carotenoid content was calculated based on the absorbances (*A*_470_, *A*_661.6_, and *A*_644.8_) of the extracts using the following equation: *c*_(x+c)_ (μg ml^–1^) = (1000*A*_470_ − 1.90 *c*_a_ − 63.14 *c*_b_)/214, in which *c*_a_ was calculated using the equation *c*_a_ (μg ml^–1^) = 11.24*A*_661.6_ − 2.04*A*_644.8_ for chlorophyll *a* content, and *c*_b_ was calculated using the equation *c*_b_ (μg ml^–1^) = 20.13*A*_644.8_ − 4.19*A*_661.6_ for chlorophyll *b* content, as described previously (Lichtenthaler and Buschmann, 2001). Values were measured based on three replicates for each plant line; the value for the VC was the average of three lines and that for the *NtCOI1*-silenced tobacco was the average of five lines.

### Starch and sugar analyses

Starch staining by I_2_/KI solution [1% (w/v) I_2_ in 3% (w/v) KI] was used to evaluate the starch content in the indicated tobacco organs: a darker colour indicates more starch contained in the tested organs. Pollen grains were released into the I_2_/KI solution by squeezing the anthers in the solution, stained for 10min, and then pipetted onto a slide and exposed to air after soaking off excess solution with blotting paper. They were photographed under a stereoscopic microscope. The ovary and corolla were separated from the flower, stained for 30min or overnight, respectively, in 10ml of I_2_/KI solution, and then photographed under a stereoscopic microscope. Fully expanded leaves of 7-week-old plants were stained directly with I_2_/KI solution for 5min and briefly rinsed with water to detect polysaccharides in the trichome secretion. Following overnight decolorizing with 95% ethanol, the leaves were stained with I_2_/KI solution for 15min and then washed with water for 5min to detect leaf starch.

Starch content in the indicated organs was quantified using a Starch Assay Kit (Sigma) according to the manufacturer’s instructions. Twenty floral nectaries, three corollas, anther wall from 30 anthers, pollen grains from 30 anthers, and 0.1g of leaf tissue were used for each measurement. To collect anther wall and pollen grains for starch measurement, anthers were put into a 2ml centrifuge tube containing 1ml water and squeezed with tweezers to completely release the pollen grains. The pollen-containing solution was transferred into a new centrifuge tube to collect pollen grains by centrifuging at 2000*g* for 6min, and the empty anther wall was collected after rinsing once with water. Values were measured based on three replicates for each plant line; the value for VC was the average of three lines, and that for the *NtCOI1*-silenced tobacco was the average of five lines. The total soluble sugar in the anther wall was measured using anthrone reagent, as described previously (Yemm and Willis, 1954).

### Nectar measurement

To collect floral nectar, the flower was laid down on a plate and two longitudinal lines were gently cut with a blade on both sides along the corolla tube to evenly divide the corolla into upper and lower portions; the upper portion was carefully peeled off. The nectar from both portions was collected in a centrifuge tube using a micropipette, and its volume was estimated with a micropipette as described previously (Carter *et al.*, 1999).

### Histological assay

To visualize the starch granules in the anther, tobacco anthers were fixed for 4h in 4% paraformaldehyde in PBS (pH 7.4) at room temperature under vacuum, dehydrated in a graded ethanol series, embedded with paraffin, sectioned to 10 μm, and dried at 42 °C. Next, the sections were deparaffinized and hydrated in a graded ethanol/water series. The sections were then stained in I_2_/KI solution for 30min, rinsed briefly with water, and photographed under a transmitted light microscope.

### Quantitative RT-PCR (qRT-PCR)

Total RNAs were extracted using TRIzol reagent (Invitrogen) according to the manufacturer’s instructions. The anther wall and pollen grains for total RNAs extraction were collected as described for starch analysis. Leaf samples for total RNA extraction were collected from fully expanded leaves of 7-week-old plants. First-strand cDNAs were synthesized using an M-MLV First Strand kit (Invitrogen), and used as templates for qRT-PCR. Reactions were performed using a Stratagene Mx3000P™ quantitative PCR system with GoTaq^®^ qPCR Master Mix (Promega).

Primers used for detecting the expression of genes are given in Supplementary Table S2 available at *JXB* online. The 18S rRNA gene was used as an internal control. The relative transcripts were obtained by calibrating the threshold cycles of genes of interest with that of the 18S rRNA gene using the equation 2^(–ΔΔ^CT^)^ as described previously (Zhang *et al.*, 2012), where *C*_T_ is the cycle number of the threshold point at which fluorescence is detectable. Transcript level values were measured based on three replicates for all experiments; the value for VC was the average of three lines and that for the *NtCOI1*-silenced tobacco was the average of five lines.

### Statistical analysis

Statistical analyses of quantitative data were performed using Microsoft Excel. Statistical significance was assessed by comparison with the control (wild-type or VC-transformed plants) using one-way analysis of variance followed by a two-tailed Student’s *t*-test: *P<0.05; **P<0.005.

### Gene accessions

The GenBank accession numbers for the *N. tabacum* genes described in this article are as follows: *NtCOI1* (AB433899), *PSY* (JF461341), *ZDS* (JF975566.1), *LYC* (X81787.1), *AGPs* (small subunit, AB018190), *SS2* (JX264160), *BAM1* (KF420482), *NtMYB305* (KC792284), *INV1* (X81834), *INV2* (AF376773), *INV3* (HM022265), *INV4* (HM022266), *INV5* (HM022267), and 18S rRNA gene (AJ236016). The tobacco *SS2*, *BAM1*, and *NtMYB305* genes were cloned according to the sequences obtained following a BLAST search against Gene-space Sequence Reads from the Tobacco Genome Initiative website (http://www.pngg.org/tgi/) as described previously (Zhang *et al.*, 2012). Sequenced fragments were used as templates to design primers for qRT-PCR.

## Results

### Male sterile phenotype of *NtCOI1*-silenced tobacco

**Fig. 1.**
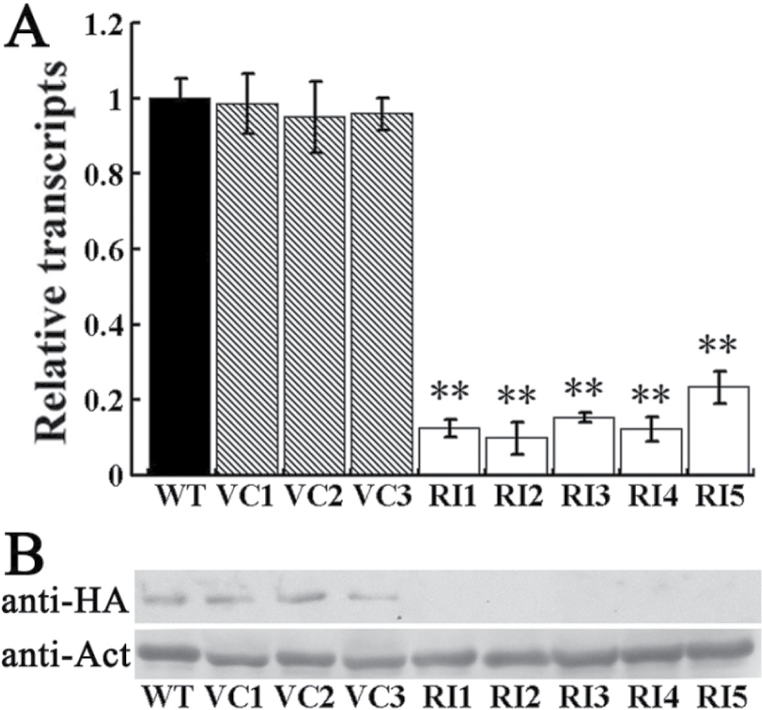
Identification of *NtCOI1*-silenced tobacco lines. (A) Relative *NtCOI1* transcript levels in *NtCOI1*-silenced (RI) and control (WT, wild type; VC, VC transformed) plants. The expression value of *NtCOI1* in WT leaves is set as 1. Results are shown as means ±standard error (SE). (B) Detection of transiently expressed HA-tagged NtCOI1 protein by western blotting. Anti-HA and anti-Act indicate blotting with antibodies against HA tag and β-actin, respectively. VC_N_ and RI_N_ indicate different independent transgenic lines. Significant differences from the data of control plants are indicated: **P<0.005 (Student’s *t*-test).

The *NtCOI1*-silenced transgenic tobacco plants were developed by RNAi-mediated gene silencing using a fragment of the *NtCOI1* gene (Supplementary Fig. S1 available at *JXB* online). Of 21 independently derived transgenic lines, seven showed >70% suppressed expression of *NtCOI1* relative to wild type, and the five lines with the greatest knockdown of *NtCOI1* transcripts were investigated in this study (Fig. 1A). To verify specific disruption of the NtCOI1 protein following RNAi-mediated gene silencing, a plasmid vector expressing an HA-tagged NtCOI1 fusion protein under the control of a 2×CaMV 35S promoter was introduced into the leaves of control and *NtCOI1*-silenced plants by agroinfiltration. Following 4 d of growth in the greenhouse, total proteins were extracted from control and agroinfiltrated leaf patches, and analysed by western blot analysis using an anti-HA antibody. NtCOI1–HA fusion protein was detected in the control leaves but was undetectable in the leaves of *NtCOI1*-silenced plants (Fig. 1B), indicating that RNAi-mediated silencing of *NtCOI1* could effectively knock down the expression of *NtCOI1*.

**Fig. 2.**
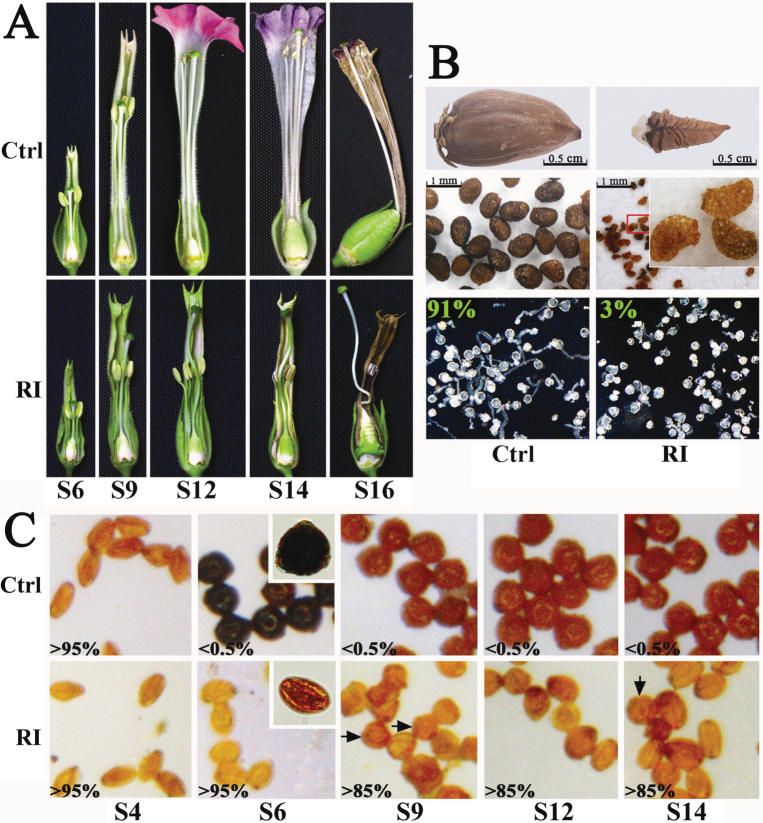
Male fertile phenotypes of *NtCOI1*-silenced (RI) and control (Ctrl) plants. (A) Floral phenotypes at indicated various floral development stages. (B) Fertilization phenotypes. Top panel, capsules; middle panel, seeds (Ctrl) and abortive seeds (RI) released from capsules; the inset shows an enlargement of the region marked by a red square; bottom panel, pollen germination assay: the percentage of normally germinated pollen grains is given. (C) Pollen staining using I_2_/KI solution. Arrows indicate round pollen grains of *NtCOI1*-silenced tobacco. The percentage of shrunk pollen grains is given in the left corner of each image. Insets in the S6 panel show enlargements of typical control and RI pollen grains. The representative control pictures were taken from VC-transformed plants.

The *NtCOI1*-silenced transgenic plants generated in this study with *NtCOI1* transcripts knocked down by > 70% were male sterile. These plants exhibited shortened stamen filaments (Fig. 2A), and had significant delayed anther dehiscence. The anthers of control plants dehisced at S10, when the corolla was still closed (Supplementary Fig. S2 available at *JXB* online), whereas the anthers of *NtCO1*-silenced plants remained indehiscent until S14, when the corolla had started to senesce and the corolla base was turning grey-brown (Fig. 2A; high-resolution images are shown in Supplementary Fig. S2 available at *JXB* online). The flowers of *NtCOI1*-silenced plants could not produce viable seed, even after manual pollination using pollen grains from the dehisced anthers of *NtCOI1*-silenced plants (Fig. 2B). Interestingly, over half of the unpollinated flowers on *NtCOI1*-silenced plants did not abscise from the plant, and their ovaries kept growing at a slow pace into small capsules that contained entirely aborted seeds (Fig. 2B), which should be empty seeds derived from the unpollinated ovules. In contrast, the flowers of *NtCOI1*-silenced plants could generate seeds after pollination using pollen grains from the control tobacco. The heterozygous T_1_ hybrid seeds produced from pollination of *NtCOI1*-silenced flowers with wild-type pollen grains were used for a JA-sensitivity assay after antibiotic resistance screening (Supplementary Fig. S3 available at *JXB* online). This revealed that *NtCOI1*-silenced seedlings were JA insensitive. The *NtCOI1*-silenced tobacco also failed to accumulate anthocyanin in the corollas, resulting in corollas with a pale green appearance (Fig. 2A; Supplementary Fig. S4 available at *JXB* online). In addition, the expression levels of anthocyanin biosynthetic genes were downregulated in the *NtCOI1*-silenced corollas (Supplementary Fig. S4 available at *JXB* online). These findings revealed that the *NtCOI1*-silenced tobacco mimicked the hallmarks of the *Arabidopsis coi1* mutant (Feys *et al.*, 1994).

The *NtCOI1*-silenced tobacco generated in this study had pollen germination defects (Fig. 2B). Pollen staining with I_2_/KI solution showed that the control pollen grains could not be stained before S4, stained dark brown at S6, and stained brown after S9. Presumably, starch in the control pollen grains was converted from amylose to amylopectin after S9, leading to the colour change of I_2_/KI-stained pollen grains from dark brown to brown (Hostettler *et al.*, 2011). However, the pollen grains of *NtCOI1*-silenced tobacco could not be stained or showed significantly less staining at all developmental stages (Fig. 2C), thus indicating lower carbohydrate deposits in the pollen. Another phenotypic difference between the pollen grains of control and *NtCOI1*-silenced plants was that pollen grains from the *NtCOI1*-silenced flower rapidly shrank following exposure to the air. From Fig. 2C, it can be seen that the control pollen grains exposed to air could maintain a round shape after S6, while the *NtCOI1*-silenced pollen grains shrank at all floral development stages, with only a few round pollen grains observed after S9 (Fig. 2C), suggesting a reduction in the deposit of substances such as polysaccharides in the *NtCOI1*-silenced pollen grains. As the *NtCOI1*-silenced tobacco plants are male sterile transgenic plants, and nearly half of their seeds produced by pollination with wild type pollen grains were non-transgenic (Supplementary Table S1 available at *JXB* online), it follows that approximately half of the pollen grains produced by these plants should be non-transgenic. However, almost all of the *NtCOI1*-silenced pollen grains were defective (Fig. 2B, C). This indicates an irregular pollen developmental environment in the anther.

### Disruption of NtCOI1 suppresses carotenoid accumulation and floral-nectar production in tobacco floral nectary

**Fig. 3.**
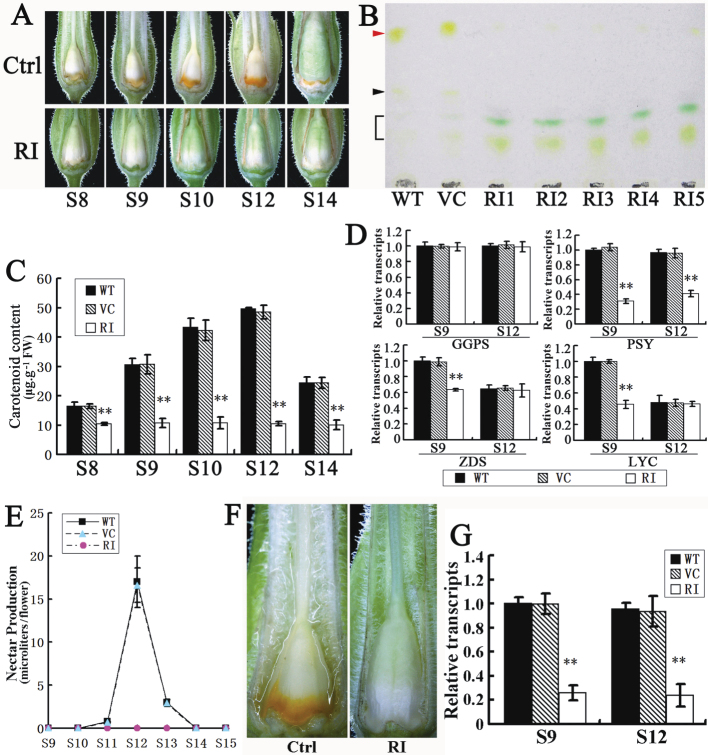
Carotenoid accumulation in floral nectaries and floral-nectar production of *NtCOI1*-silenced (RI) and control (Ctrl) plants. (A) Visualization of carotenoid in the nectaries at the indicated floral development stages. (B) TLC assay of carotenoids in nectaries at S12. The red arrowhead indicates β-carotene spots and the black arrowhead indicates an unidentified pigment. The black bracket indicates chlorophylls. WT, wild type; VC, VC transformed. RI_N_ indicates independent transgenic lines. (C) Quantification of carotenoid in nectaries at the indicated stages. Results are shown as means ±SE. (D) Relative transcript levels of genes involved in carotenoid biosynthesis in nectaries at the indicated stages. *GGPS*, geranylgeranyl diphosphate synthase. The expression value of each gene in S9 WT nectaries is set as 1. Results are shown as means ±standard deviation (SD). (E) Floral-nectar content at indicated stages. Nectar volumes are based on measurement of five flowers from each plant line. Results are shown as means ±SD. (F) Visualization of floral nectar at S12. (G) The relative transcript levels of the nectarin gene *Nec1* in nectaries at the indicated floral development stages. The expression value of *Nec1* in S9 WT nectary is set as 1. The representative Ctrl pictures were taken from the VC plant. Significant differences from the data of control plants are indicated: **P<0.005 (Student’s *t*-test). Results are shown as means ±SD.

During floral development, the control floral nectary turned orange during S9–S14 (Fig. 3A) due to carotenoid accumulation (Horner *et al.*, 2007). However, no visible orange pigmentation was observed in the floral nectary of *NtCOI1*-silenced tobacco (Fig. 3A). A TLC assay of pigments in S12 floral nectaries revealed that the predominant pigment (the front spot on the plate) in tobacco cv. TN90 was β-carotene (Supplementary Fig. S5A available at *JXB* online), consistent with a previous report on ornamental tobacco (Horner *et al.*, 2007). The ovary of control plant was milky-yellow with an orange floral nectary at S12, whereas the ovary of *NtCOI1*-silenced plants was green with a pale green floral nectary at the same stage (Fig. 3A), suggesting a higher chlorophyll level in the *NtCOI1*-silenced floral nectary. The TLC assay revealed that β-carotene accumulation was markedly lower in the floral nectary of *NtCOI1*-silenced tobacco than in the control, whereas chlorophylls were at higher levels (Fig. 3B). This finding indicated irregular chlorophyll synthesis in the floral nectary of *NtCOI1*-silenced tobacco during flower development. Of interest is the presence of an unidentified yellow pigment that was only observed in the control floral nectaries (Fig. 3B). Quantification of carotenoids from S8–S14 floral nectaries revealed a shift in content in the control but not the *NtCOI1*-silenced nectaries (Fig. 3C). Transcriptional analyses of a set of carotenoid synthetic genes, whose expression displayed different degrees of dependence on floral development stages in wild-type floral nectary (Supplementary Fig. S5B available at *JXB* online), revealed that expression of phytoene synthase (*PSY*), ζ-carotene desaturase (*ZDS*), and lycopene cyclase (*LYC*) was suppressed in the *NtCOI1*-silenced floral nectaries compared with the control at the carotenoid accumulation stage (S9), and no obvious differences in the gene transcripts were observed at the completion stage of carotenoid accumulation (S12), with the exception of *PSY* (Fig. 3D).

The *NtCOI1*-silenced tobacco did not produce floral nectar. In wild-type flowers, a small volume of floral nectar was observed at S11, and the volume rapidly increased to approximately 20 μl per flower at S12. After S12, the floral nectar quickly decreased to an unobservable level (Fig. 3E). However, no floral nectar was observed in the flowers of *NtCOI1*-silenced plants at any floral development stage (Fig. 3E). In longitudinally cut flowers, a noticeable quantity of floral nectar (the reflective fluid) was observed in the control corolla tubes, while none was visible in the *NtCOI1*-silenced corollas (Fig. 3F). The expression of *Nectarin 1* (*Nec1*), whose protein product is secreted with floral nectar and functions in the nectar redox cycle to produce H_2_O_2_ (Carter and Thornburg, 2000; Carter *et al.*, 2007; Liu and Thornburg, 2012), was greatly downregulated in the *NtCOI1*-silenced floral nectaries (Fig. 3G).

**Fig. 4.**
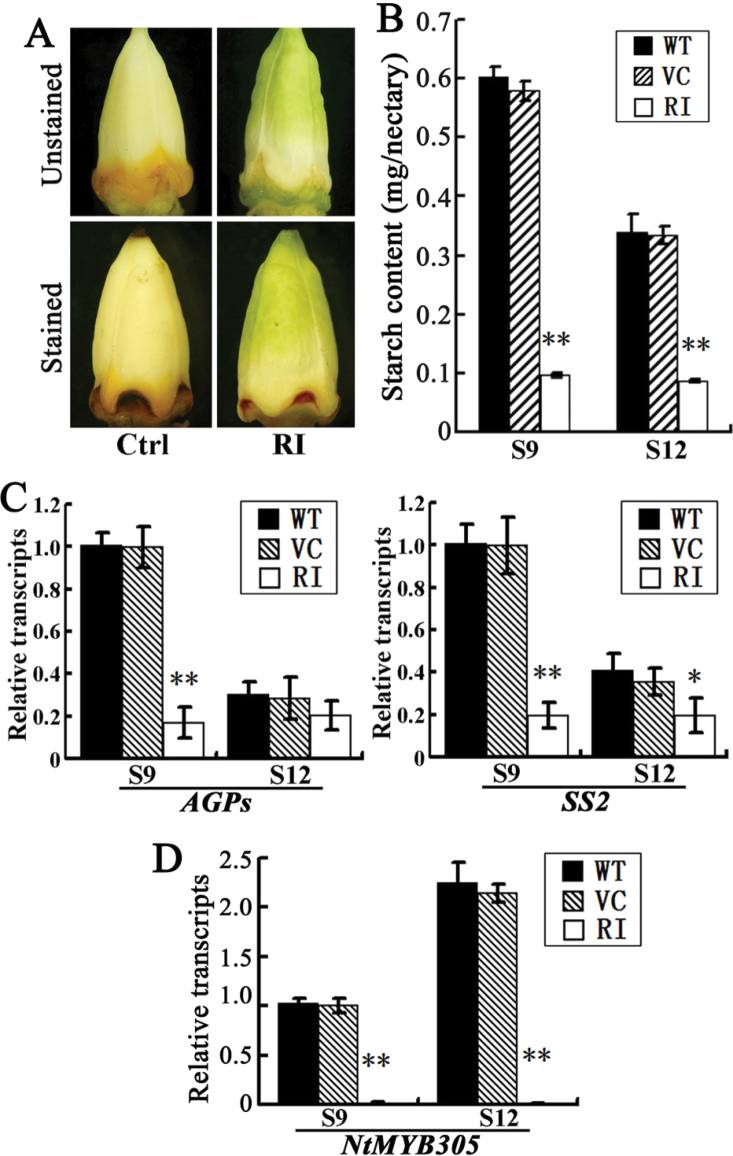
Starch synthesis and transcription of *NtMYB305* in the floral nectaries of *NtCOI1*-silenced (RI) and control (Ctrl) plants. (A) Visualization of nectary starch at S12 by I_2_/KI staining. The representative Ctrl picture was taken from VC-transformed plants. (B) Starch content in nectaries at the indicated floral development stages. WT, wild type. Error bar, mean ± SE. (C) Relative expression levels of starch metabolic genes in nectaries at indicated floral development stages. Results are shown as means ±SD. The expression value of each gene in the S9 WT nectaries is set as 1. (D) Relative *NtMYB305* transcript levels in nectaries at indicated floral development stages. Results are shown as means ±SD. The expression value of *NtMYB305* in S9 WT nectary is set as 1. Significant differences from the data of control plants are indicated: *P<0.05; **P<0.005 (Student’s *t*-test). (This figure is available in colour at *JXB* online.)

Nectar secretion is closely correlated with starch metabolism (Horner *et al.*, 2007), the source of nectar sugar. To compare starch metabolism differences, floral nectary starch was visualized by I_2_/KI staining. The control nectary gland was stained dark brown, while that of the *NtCOI1*-silenced plants was light brown with a smaller stained area (Fig. 4A), thus indicating reduced starch content in the floral nectaries of *NtCOI1*-silenced plants. Measurements revealed that the starch content in the *NtCOI1*-silenced nectaries was less <10% of the control plants at S9 (starch metabolism shifting from anabolism to catabolism; Liu and Thornburg, 2012) and S12 (flower corolla fully opened; Drews *et al.*, 1992) (Fig. 4B). A transcriptional assay of starch metabolic genes revealed that expression of ADP-glucose pyrophosphorylase (small subunit) (*AGPs*) and soluble starch synthase II (*SS2*) was markedly downregulated in the floral nectary of *NtCOI1*-silenced tobacco at S9 compared with the control, and their lower expression levels were maintained at S12 (Fig. 4C).

The above findings indicated that NtCOI1 functions similarly to ornamental tobacco MYB305 in regulating carotenoid accumulation and floral-nectar production (Carter *et al.*, 2007; Liu and Thornburg, 2012), suggesting NtCOI1 involvement in controlling starch metabolism. To reveal the relationship between NtCOI1 and MYB305 in the regulation of floral-nectar production and starch metabolism in the floral nectaries, we cloned the tobacco (cv. TN90) MYB305 orthologue (designated as NtMYB305; see Supplementary Fig. S6 available at *JXB* online for sequence alignment), and analysed its transcript levels in the floral nectaries of *NtCOI1*-silenced tobacco. The results revealed that silencing of *NtCOI1* severely downregulated expression of *NtMYB305* in floral nectaries at S9 and S12 (Fig. 4D). Thus, these findings suggest that NtCOI1 functions upstream of NtMYB305 in controlling primary carbohydrate metabolism in the floral nectary, and provide important clues to uncover the underlying molecular mechanism of COI1-regulated pollen development.

### Silencing of NtCOI1 alters starch accumulation in organs correlated with pollen development

**Fig. 5.**
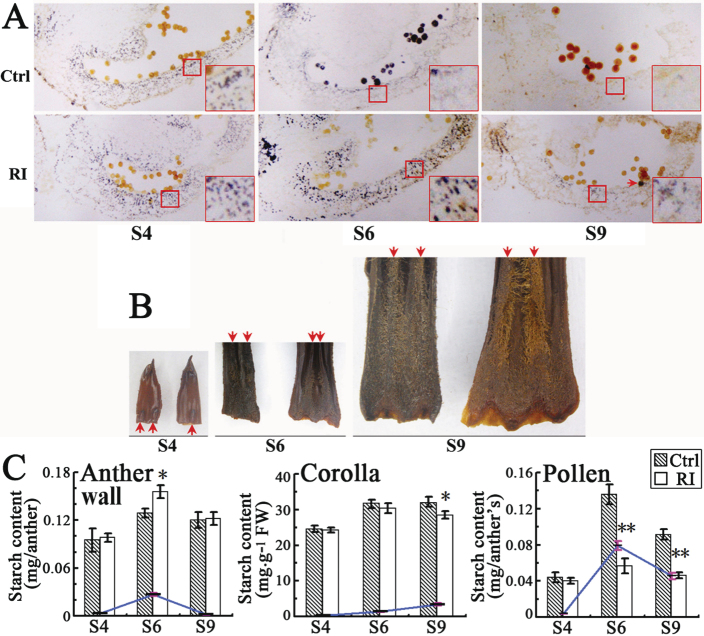
Starch accumulation in the anther wall, pollen, and corolla of *NtCOI1*-silenced (RI) and control (Ctrl) plants. (A) I_2_/KI staining of the anther wall and pollen. The inset in each panel is an enlargement of the region marked by the smaller red square. The red arrow in the S9 RI anther wall indicates a black-stained pollen. (B) I_2_/KI staining of RI (right) and control (left) corollas at the indicated floral development stages. Red arrows indicate the stamen filaments. (C) Starch content in the anther wall, corolla, and pollen. Coloured curves indicate the change of the absolute values of starch content differences in the control and RI organs at indicated floral development stages. Error bar, mean ± SE. The representative control pictures and data were taken from the VC plant. Significant differences from the data of control plants are indicated: *P<0.05; **P<0.005 (Student’s *t*-test).

**Fig. 6.**
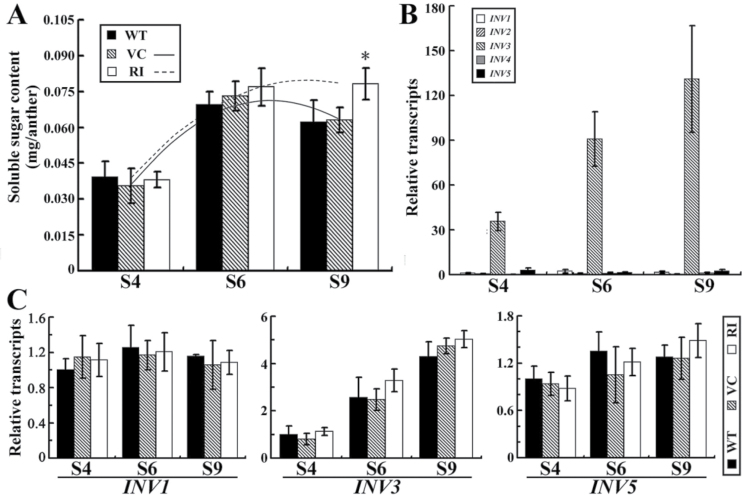
Analyses of total soluble sugar and invertase gene expression in the anther wall. (A) The amount of total soluble sugars in the anther wall at the indicated floral development stages. WT, wild type; VC, VC-transformed; RI, *NtCOI1* silenced. Trendlines indicate change curves in VC and RI anthers over the indicated floral development stages. (B) The expression patterns of invertase genes in the wild-type anther wall at the indicated floral development stages. *INV1–5* indicates cell-wall invertase genes 1–5, respectively. The expression value of *INV1* in the S4 anther wall was set as 1. (C) Relative transcript levels of invertase genes in the anther wall at the indicated floral development stages. Significant difference from the data of control plants are indicated: *P<0.05 (Student’s *t*-test).

To reveal changes in primary metabolism that might affect pollen development in the *NtCOI1*-silenced tobacco, starch metabolism was examined in the anther as well as in the corolla, because stamen filaments of tobacco fuse with the corolla (Fig. 5B) and rapid substance exchange between them is allowed. Visualization of starch granules in anthers was performed by a histological assay (Fig. 5A). I_2_/KI-stained anther sections revealed that starch granules were of a similar level in the control and *NtCOI1*-silenced anther wall at S4, while levels reduced in the control anther wall at S6 but increased to a much higher level in the *NtCOI1*-silenced anther wall (Fig. 5A). Starch granules were not observed in the control but were clearly visible in the *NtCOI1*-silenced anther wall at S9 (Fig. 5A). As with the direct pollen staining described above (Fig. 2C), in the I_2_/KI-stained anther sections, the control pollen grains were stained black at S6 and brown at S9, while the *NtCOI1*-silenced pollen grains had no staining or less staining at S6 and S9, with quite a few stainable pollen grains in the section of *NtCOI1*-silenced anther at S9 (Fig. 5A). These findings revealed an interesting phenomenon where the *NtCOI1*-silenced anther wall accumulated a high level of starch granules at S6 but the pollen grains were not stainable with iodine, implying irregular starch catabolism in the *NtCOI1*-silenced anther wall. Furthermore, I_2_/KI staining revealed no significant difference between the control and *NtCOI1*-silenced corollas at S4, whereas a large colour difference between them was seen at S9, when the control pollen grains had already matured (Fig. 5B). This does not support the suggestion that starch reduction caused by *NtCOI1*-silencing in the corolla necessarily results in a starch deficit in the anther. Measurement of starch content in these organs showed consistent results to I_2_/KI staining (Fig. 5C). Sugar measurements revealed that the amount of total soluble sugars in the *NtCOI1*-silenced anther wall was similar to that of the control at S4 and S6 but at a slightly higher level at S9 (Fig. 6A), indicating an altered starch metabolic process in the *NtCOI1*-silenced anther wall. To determine whether this was caused by the decrease in sink strength, we tested the expression of anther-specific cell-wall invertases (Goetz *et al.*, 2001; Engelke *et al.*, 2010), which are sink-strength regulators controlling the conversion of sucrose and hexoses (Asthir and Singh, 1995; Fernie *et al.*, 2002). The transcriptional analyses showed that cell-wall invertase 3 (*INV3*) was the most abundantly expressed cell-wall invertase gene in the anther wall of tobacco cv. TN90 (Fig. 6B), with a higher expression level seen in the anther wall at pollen maturation stages S6 and S9. However, further studies found no significant decrease in the transcript levels of invertase genes in the *NtCOI1*-silenced anther wall (Fig. 6C). Therefore, this does not support the hypothesis that *NtCOI1*-silencing may reduce anther sink strength. Taken together, these findings provide further evidence of NtCOI1 involvement in the regulation of primary carbohydrate metabolism.

### Transcription profiles of *NtMYB305* and starch metabolic genes are altered in the anther wall, corolla, and pollen of *NtCOI1*-silenced tobacco

**Fig. 7.**
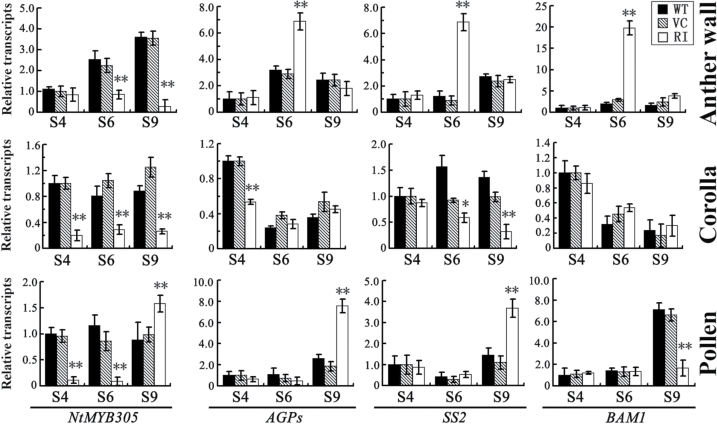
Transcriptional analyses of *NtMYB305* and starch metabolic genes in the anther wall, corolla, and pollen. WT, wild type; VC, VC transformed; RI, *NtCOI1* silenced. The transcription of each gene in S4 wild-type organs was set as 1. Results are shown as means ±SD. Significant differences from the data of control plants are indicated: *P<0.05; **P<0.005 (Student’s *t*-test).

To determine the molecular mechanism by which NtCOI1 regulates starch metabolism in pollen-development-correlated organs, the transcription profiles of *NtMYB305* and starch metabolic genes were analysed in the anther wall, corolla, and pollen. Fig. 7 shows that the expression level of *NtMYB305* increased in the control anther wall from S4 to S9, but no increase was observed in the *NtCOI1*-silenced anther wall. The transcription of the starch metabolic genes *AGPs*, *SS2*, and β-amylase 1 (*BAM1*) increased steadily in the control anther wall from S4 to S9 but exhibited a dramatic increase in the *NtCOI1*-silenced anther wall at S6 (Fig. 7). This is consistent with the abnormal accumulation of starch granules in the anther wall (Fig. 5A). The expression levels of *NtMYB305* were downregulated in the corolla of *NtCOI1*-silenced plants, but expression of the analysed starch metabolic genes did not show a significant decrease, with the exception of *SS2* at S9 (Fig. 7). The transcription of *NtMYB305* and starch metabolic genes displayed interesting profiles in the pollen of *NtCOI1*-silenced plants. The transcription of *NtMYB305* was obviously downregulated in the pollen of *NtCOI1*-silenced plant at S4 and S6 but exhibited a dramatic increase at S9, which was followed by an increase in the transcript levels of *AGPs* and *SS2* (Fig. 7). These findings support the hypothesis that NtCOI1 functions upstream of NtMYB305 in regulating primary metabolism, and imply that starch metabolism may undergo different regulatory patterns in the anther wall, corolla, and pollen.

### Leaf primary metabolism is changed in *NtCOI1*-silenced tobacco

**Fig. 8.**
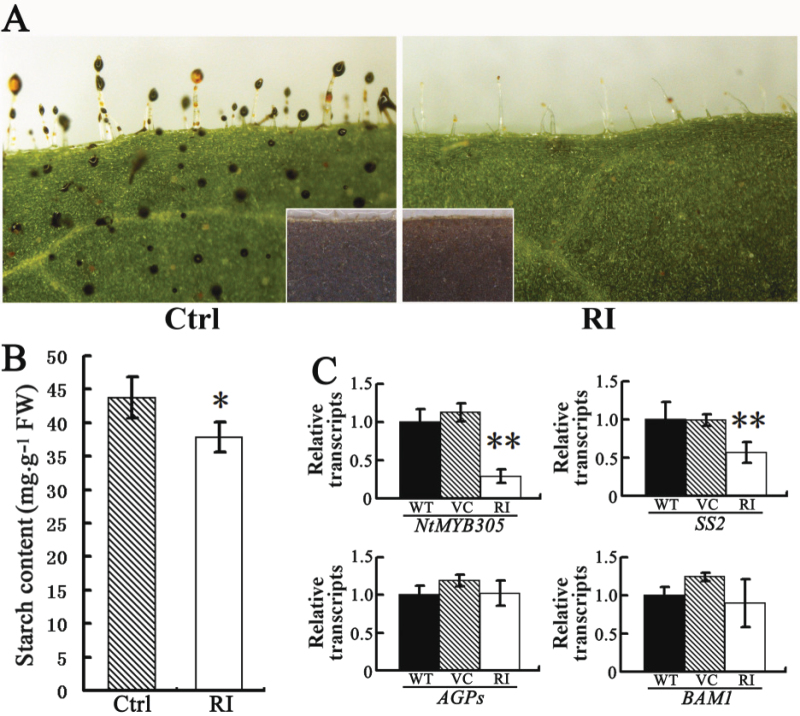
Leaf morphology of *NtCOI1*-silenced (RI) tobacco. (A) I_2_/KI staining of leaves. Insets show leaves stained after ethanol decolorization. The representative control (Ctrl) pictures were taken from VC-transformed plant. (B). Starch content in leaves. Results are shown as means ±SE. The representative Ctrl data were taken from the VC plant. (C) Relative expression levels of *NtMYB305* and starch metabolic genes in leaves. WT, wild type. The transcription of each gene in WT leaves was set as 1. Results are shown as means ±SD. Significant differences from the data of control plants are indicated: *P<0.05; **P<0.005 (Student’s *t*-test).

The primary metabolic changes observed in the flower of *NtCOI1*-silenced tobacco stimulated us to determine the influences of *NtCOI1* silencing on leaf primary metabolism. Interestingly, we observed a striking difference between the trichomes of control and *NtCOI1*-silenced tobacco leaves (Fig. 8A) following a brief iodine staining. The control trichome heads were easily stainable by I_2_/KI solution, indicating an abundance of polysaccharides, whereas the trichome heads of *NtCOI1*-silenced leaves were nearly unstained. Furthermore, the iodine-stainable part of the control trichome could easily be washed off by ethanol (Supplementary Fig. S7 available at *JXB* online), suggesting that it is a droplet of trichome secretion that contains polysaccharides and essential oils (Werker, 1993). Therefore, these results suggested an alteration in trichome-secretion-related carbohydrate metabolism in the *NtCOI1*-silenced plant. Measurement of leaf starch showed that silencing of *NtCOI1* reduced the leaf starch content by ~12% (Fig. 8B). However, iodine staining of the ethanol-decolorized leaves showed that the *NtCOI1*-silenced leaf was slightly less stained than the control (Fig. 8A). The transcriptional analysis revealed that the expression of *NtMYB305* and the starch anabolic gene *SS2* was downregulated in the *NtCOI1*-silenced leaf, but no obvious transcriptional changes in *AGPs* and *BAM1* were observed (Fig. 8C), thus indicating different regulatory patterns for starch metabolic genes in the leaf. These observations suggest that silencing of *NtCOI1* influenced the primary carbohydrate metabolism in vegetative tissue, although no other apparent morphological changes were observed in this type of tissue.

## Discussion

COI1 is a co-receptor for JA-Ile, and regulates male fertility, secondary metabolism, and JA responses (Feys *et al.*, 1994; Xie *et al.*, 1998). In this study, we developed *NtCOI1*-silenced tobacco that exhibited male sterility, JA insensitivity, and loss of anthocyanin, which are the prominent characteristics of *coi1* mutant (Feys *et al.*, 1994; Xie *et al.*, 1998). We demonstrated that silencing of *NtCOI1* altered primary carbohydrate metabolism in tobacco, and influenced the primary-metabolism-correlated physiological processes, including pollen development, floral-nectar production, carotenoid accumulation in floral nectary, and trichome secretion, and changed the transcription profiles of *NtMYB305* and a set of starch metabolic genes. We have therefore demonstrated a role for NtCOI1 in the regulation of primary carbohydrate metabolism.

Pollen grains in plants with defective COI1 are non-viable (Feys *et al.*, 1994; Xie *et al.*, 1998; Li *et al.*, 2004; Paschold *et al.*, 2007; Song *et al.*, 2011). The *NtCOI1*-silenced tobacco consistently showed a male sterile phenotype. Nearly half of the *NtCOI1*-silenced tobacco progenies produced from cross-pollination with wild-type pollen grains were non-transgenic (Supplementary Table S1 available at *JXB* online), indicating that approximately 50% of pollen grains produced by these plants should be non-transgenic. However, starch metabolism was suppressed in almost all of the *NtCOI1*-silenced pollen grains, implying abnormal starch metabolism in the anther wall that provides the environment for pollen development. Although the stamen filaments of tobacco are fused to the corolla, the *NtCOI1*-silenced corollas only showed relatively low starch content at S9 when mature pollen grains had already formed and corolla anthocyanin accumulation had started in the control, implicating that the decrease in corolla starch content may correlate with the development of corolla but not of pollen. Further studies revealed an abnormal accumulation of starch granules in the *NtCOI1*-silenced anther wall at S6, whereas no starch accumulation was observed in the *NtCOI1*-silenced pollen at this stage. During floral development in tobacco, S6 is after microspore separation from the tetrad (at S2) and tapetum degeneration (at S3–S4), and microspores are nourished by locular fluid in the anther at this stage (Koltunow *et al.*, 1990; Drews *et al.*, 1992; Pacini *et al.*, 2006). Therefore, altered starch metabolism in the *NtCOI1*-silenced anther wall at S6 may be a critical factor affecting pollen development. Tapetum, an important source of bioactive gibberellins in flower (Kaneko *et al.*, 2003), plays a major role in pollen development (Powell *et al.*, 2012). The complete pollen abortion in *NtCOI1*-silenced tobacco implies a possibility of tapetum dysfunction. Formation of microspores in the *NtCOI1*-silenced tobacco showed no obvious differences to the control, and a previous study in *Arabidopsis* demonstrated that the *coi1* mutant produces normal tetrads and microspores (Devoto *et al.*, 2002). Therefore, the regulation of tapetum behaviour by COI1 remains to be determined. Efforts to determine the sink-strength alteration in the *NtCOI1*-silenced anther found no significant decrease in the expression of cell-wall invertase genes that control the conversion of sucrose and hexoses (Asthir and Singh, 1995; Fernie *et al.*, 2002), suggesting that *NtCOI1* silencing may not reduce the sink strength in the anther. The *NtCOI1*-silenced anther wall retained a slightly higher level of total soluble sugars at S9, resulting in an altered trendline in the total soluble sugar level (Fig. 6A). This fact also suggests that *NtCOI1* silencing does not reduce the sink strength in the anther, as previous studies suggest that invertases do not control the amount of total soluble sugars but regulate the ratio of sucrose to hexose (Zrenner *et al.*, 1996). Presumably, the change in total soluble sugar content was a result of altered starch metabolic processes in the *NtCOI1*-silenced anther wall.

The *NtCOI1*-silenced tobacco exhibited loss of floral nectar and suppression of carotenoid and starch accumulation in the floral nectary, which is similar to the observations in *MYB305*-silenced ornamental tobacco as introduced above (Sablowski *et al.*, 1994; Liu *et al.*, 2009; Liu and Thornburg, 2012). The fertility of *MYB305*-silenced ornamental tobacco is unknown, but it is an orthologue of *Arabidopsis MYB21* and *MYB24* genes known to regulate male fertility (Chung and Howe, 2009; Song *et al.*, 2011). *MYB305*-silenced ornamental tobacco exhibited a ‘juvenile’ corolla (Liu and Thornburg, 2012) similar to that of the *NtCOI1*-silenced tobacco (Fig. 2A). Like NtCOI1, MYB305 also regulates the expression of anthocyanin biosynthetic genes (Liu *et al.*, 2009), although no anthocyanin accumulation is observable in the corolla of ornamental tobacco (*Nicotiana langsdorffii*×*Nicotiana sanderae*), which possesses an unpigmented corolla (Liu and Thornburg, 2012). These findings suggest a functional overlap between NtCOI1 and MYB305 (Liu and Thornburg, 2012). The difference between these two regulators is that, while floral nectar is weakly produced by the *MYB305*-knockdown plant, it is completely abolished in the *NtCOI1*-silenced one. Furthermore, transcription of *NtMYB305* was significantly downregulated in the floral nectary of *NtCOI1*-silenced tobacco. This suggests that NtCOI1 functions upstream of NtMYB305 in regulating primary metabolism, and also provides important clues for dissection of the primary metabolism mechanism underlying pollen development in the *NtCOI1*-silenced tobacco.

Transcriptional analyses revealed that *NtCOI1* silencing altered the transcription profiles of primary metabolic genes in pollen-development-correlated organs. The expression of *NtMYB305* was suppressed in the *NtCOI1*-silenced anther wall at S6 and S9; however, there was a dramatic increase in the expression of starch metabolic genes at S6, while those in the control anther wall showed a steady increase from S4 to S9. This is consistent with the high-level accumulation of starch granules observed in the *NtCOI1*-silenced anther wall. The transcription of *NtMYB305* in *NtCOI1*-silenced corollas was also suppressed, but expression of starch metabolic genes did not show a significant decrease with the exception of *SS2* at S9, implying different regulatory patterns in the corolla and anther wall. It is interesting that transcription of *NtMYB305* was downregulated at S4 and S6 but surged at S9 in the *NtCOI1*-silenced pollen, and similar expression patterns of the starch anabolic genes *AGPs* and *SS2* were observed. This phenomenon might reflect alternative regulation of *NtMYB305* by NtCOI1, or be the result of altered physiological processes in the pollen grains. The CaMV 35S promoter is active in pollen (Wilkinson *et al.*, 1997); thus, silencing of *NtCOI1* by the 2×CaMV 35S promoter-driven RNAi should have direct effects on the physiological processes in the pollen. The altered expression of *NtMYB305* and starch metabolic genes, especially in the anther wall, may act as an important factor affecting pollen development in *NtCOI1*-silenced tobacco. These findings support NtCOI1 functioning upstream of NtMYB305 to regulate primary metabolism. Analysis of the spatial expression pattern of *NtCOI1* showed that it was expressed in all tested tobacco tissues, including the anther wall and pollen (Supplementary Fig. S8 available at *JXB* online), revealing the capability of NtCOI1 to modulate the metabolic processes in these organs. The function of NtCOI1 in regulating the AGPase gene, which exerts significant control over starch synthesis (Stitt and Zeeman, 2012), indicates its ability to coordinate overall starch metabolism.

This study provides important evidence of the JA-signalling pathway in coordinating primary and secondary metabolism, as indicated previously (van der Fits and Memelink, 2000). JA has been known to affect carotene synthesis for decades (Saniewski and Czapski, 1983; Pérez *et al.*, 1993; Rudell and Mattheis, 2008), but less is known about its underlying molecular mechanisms. Studies on β-carotene formation in tobacco floral nectaries established that sugars from starch catabolism enter the β-carotene biosynthetic pathway through the MEP (2-C-methyl-d-erythritol-4-phosphate) pathway and act as precursors of β-carotene (Horner *et al.*, 2007). Silencing of *NtCOI1* abolished carotenoid accumulation, greatly reduced starch content, and suppressed *NtMYB305* expression in the floral nectary, providing critical molecular evidence for JA in mediating primary metabolism and carotenoid synthesis. A JA-responsive ERF transcription factor ORCA3 was shown to coordinate primary and secondary metabolism in controlling the synthesis of terpenoid indole alkaloids (van der Fits and Memelink, 2000), one of whose precursors is synthesized through the carotenoid synthetic pathway (van der Fits and Memelink, 2000). However, ORCA3 did not regulate the carotenoid biosynthetic enzyme geranylgeranyl diphosphate synthase (GGPS) (van der Fits and Memelink, 2000), whose expression is downregulated by silencing of *NtCOI1*, suggesting that NtCOI1 plays a role in ORCA3-mediated metabolism. Furthermore, the alteration in anthocyanin synthesis and starch metabolism in the *NtCOI1*-silenced corolla indicates that NtCOI1 may coordinate primary and secondary metabolism in the corolla.

An alteration in primary carbohydrate metabolism was observed in the leaves of *NtCOI1*-silenced tobacco, with trichome secretion being almost completely lost. Regulation of trichome initiation by JA-signalling components has been well documented (Li *et al.*, 2004; Yoshida *et al.*, 2009; Qi *et al.*, 2011). The finding that NtCOI1 regulates trichome secretion has deepened the current understanding of the JA-signalling pathway in regulating trichome development, and provides evidence of NtCOI1 involvement in regulating primary carbohydrate metabolism in vegetative tissue. Furthermore, recent studies have provide evidence for the involvement of both carbohydrate metabolic and photosynthetic genes in plant nectar production (Kram *et al.*, 2009; Hampton *et al.*, 2010; Lüttge, 2013; Orona-Tamayo *et al.*, 2013). Hence, a role for NtCOI1 in regulating photosynthesis is indicated. Our findings suggest that starch metabolic genes are differentially regulated in various plant tissues by NtCOI1, indicating the importance of the JA-signalling pathway in coordinating overall primary metabolism in plants. Taken together, this study establishes that NtCOI1, working upstream of MYB regulators, regulates primary carbohydrate metabolism in tobacco. In addition, our work provides an important starting point to unravel the core JA-signalling components in coordinating primary metabolism and correlated physiological processes in plants.

## Supplementary data

Supplementary data are available at *JXB* online.

Supplementary Fig. S1. Schematic diagram of the *NtCOI1* region used for RNAi-mediated gene silencing.

Supplementary Fig. S2. Flower morphology of tobacco cv. TN90 at different floral development stages.

Supplementary Fig. S3. JA-sensitivity assay for *NtCOI1*-silenced tobacco seedlings.

Supplementary Fig. S4. Silencing of *NtCOI1* downregulates anthocyanin synthesis in tobacco corolla.

Supplementary Fig. S5. Carotenoid assay, and transcripts of carotenoid synthetic genes in floral nectary.

Supplementary Fig. S6. Alignment of ornamental tobacco *MYB305* with its commercial tobacco orthologue *NtMYB305*.

Supplementary Fig. S7. Morphology of unstained trichomes.

Supplementary Fig. S8. Spatial expression patterns of *NtCOI1*.

Supplementary Table S1. Screening of hygromycin-resistant progenies from *NtCOI1*-silenced tobacco produced by pollination with wild-type pollen grains.

Supplementary Table S2. Oligonucleotide primers used for qRT-PCR.

Supplementary Method. Anthocyanin quantification.

Supplementary Data

## Abbreviations:

CaMV: cauliflower mosaic virus
HA: haemagglutinin
JA: jasmonate
JA-Ile: jasmonoyl-l-isoleucine
qRT-PCR: quantitative reverse transcription-PCR
RNAi: RNA interference
SE: standard error
SD: standard deviation
TLC: thin layer chromatography
VC: vector control.
