# Supplementary material for: The F-box protein COI1 functions upstream of MYB305 to regulate primary carbohydrate metabolism in tobacco (Nicotiana tabacum L. cv. TN90)
Source: J Exp Bot. 2014 Mar 6;65(8):2147–60. doi: 10.1093/jxb/eru084 (PMC3991746; doi:10.1093/jxb/eru084)
Supplement: Supplementary Data [file supp_65_8_2147__index.html]

The F-box protein COI1 functions upstream of MYB305 to regulate primary carbohydrate metabolism in tobacco (Nicotiana tabacum L. cv. TN90) — Supplementary Data 

# The F-box protein COI1 functions upstream of MYB305 to regulate primary carbohydrate metabolism in tobacco (*Nicotiana tabacum* L. cv. TN90)

## Supplementary Data

Data files

**Files in this Data Supplement:**

- Supplementary Data - Supplementary Data
