# Supplementary material for: The F-box protein COI1 functions upstream of MYB305 to regulate primary carbohydrate metabolism in tobacco (Nicotiana tabacum L. cv. TN90)
Source: J Exp Bot. 2014 Mar 6;65(8):2147–60. doi: 10.1093/jxb/eru084 (PMC3991746; doi:10.1093/jxb/eru084)
Supplement: Supplementary Data [file supp_eru084_eru084_New_Supplementary_Data.pdf]

SUPPLEMENTARY DATA

Supplementary Figures:

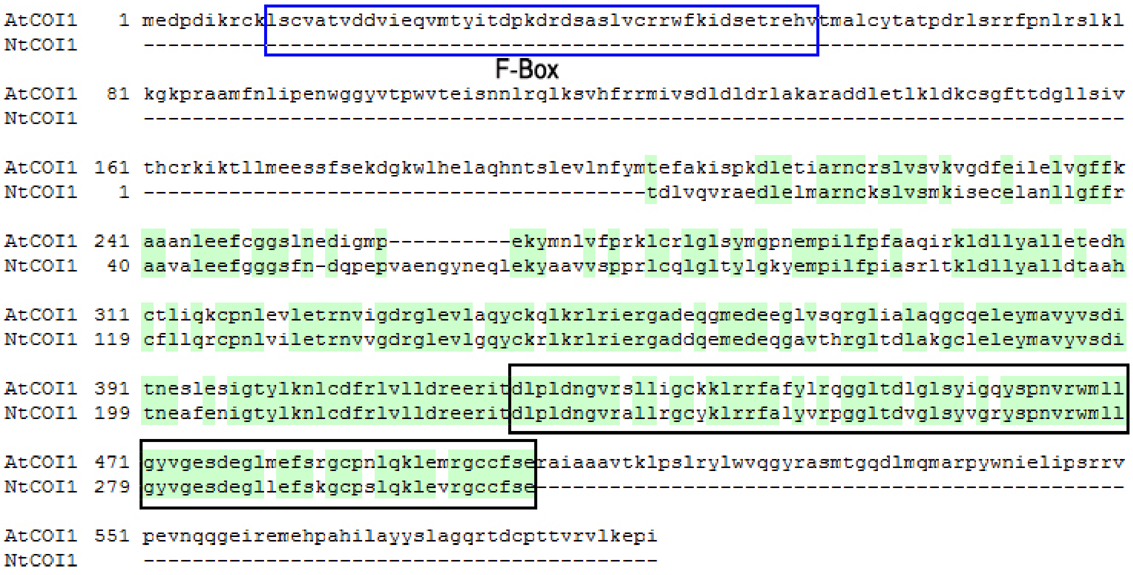

Fig. S1. Schematic diagram of the NtCOI1 region used for RNAi-mediated gene silencing. The RNAi vector was constructed using the *NtCOI1* cDNA fragment corresponding to the black boxed region in the alignment of NtCOI1 (AB433899) with AtCOI1 (AF036340). The blue boxed region indicates the F-box of AtCOI1.

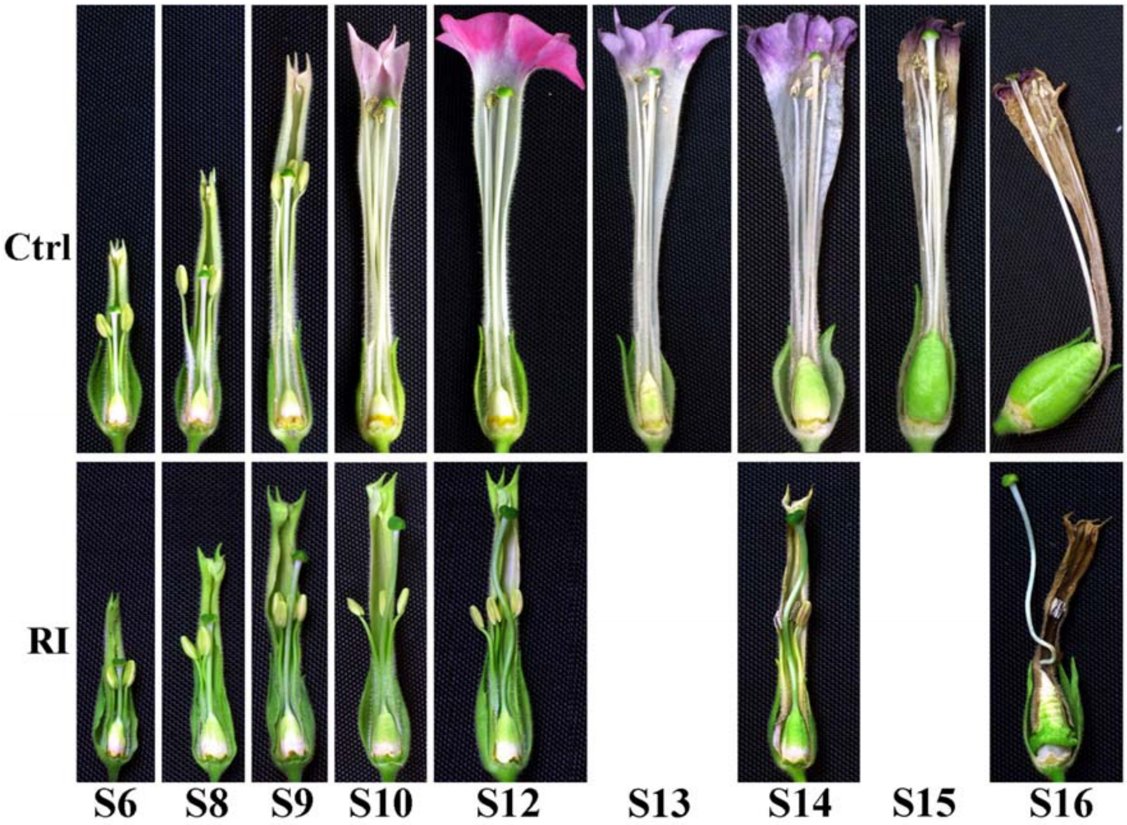

Fig. S2. Flower morphology of tobacco cv. TN90 (Ctrl) at different floral development stages. The *NtCOI1*-silenced (RI) flowers at corresponding floral development stages are given for phenotype comparison.

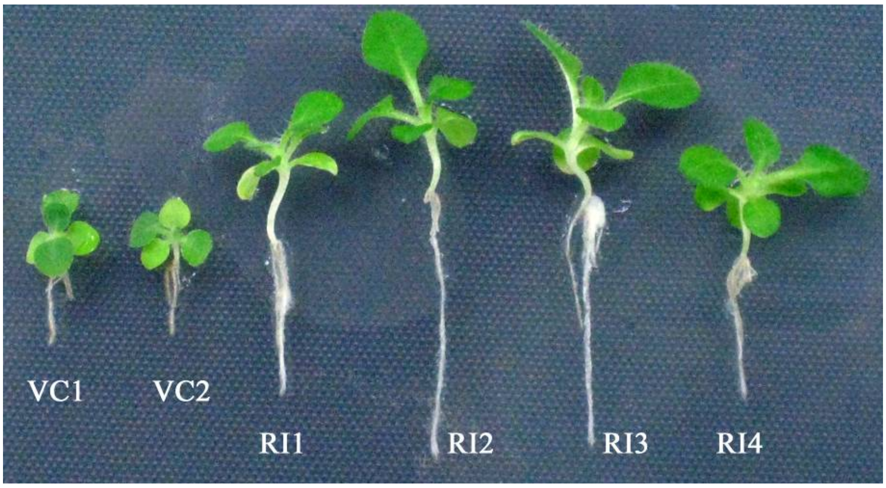

Fig. S3. JA-sensitivity assay for *NtCOI1*-silenced tobacco seedlings.

Due to the male infertility of *NtCOI1*-silenced (RI) plants, their T_1 seeds were generated by pollination using pollen grains from wild type plants. The prescreened hygromycin-resistant seedlings of RI and control vector transformed (VC) plants were used in this assay. Images of seedlings were taken after 20 days of growth on 1/2 MS agar plate containing 5 μM MeJA. VC_N or RI_N indicate different independent transgenic lines.

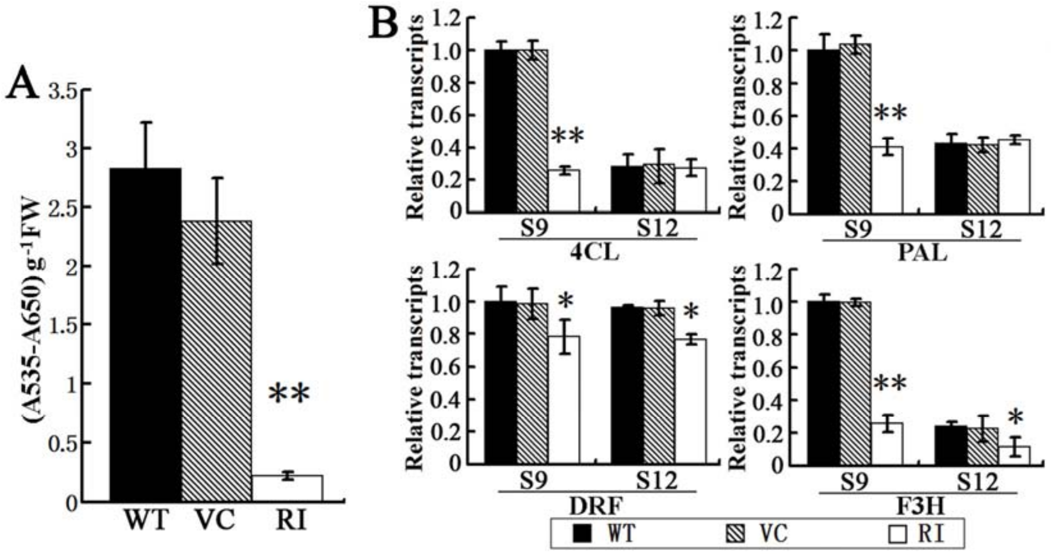

Fig. S4. Silencing of *NtCOI1* down-regulated anthocyanin synthesis in tobacco corolla.

(A) Comparison of anthocyanin content in the corollas at S12. Anthocyanin in corolla was quantified as described in Supplementary Methods. WT, wild type; VC, control vector transformed; RI, *NtCOI1*-silenced. (B) The relative transcript levels of anthocyanin synthetic genes in corollas at indicated floral development stages. Gene names and NCBI accessions: *4CL* (4-coumarate: coenzyme A ligase; U50845), *PAL* (phenylalanine ammonia-lyase; M84466.1), *F3H* (flavanone 3-hydroxylase; AF036093), *DRF* (dihydroflavonol 4-reductase; AB289448.1). The values for anthocyanin content and gene expression were determined based on three replicates, the value for VC is the average of three lines, and that for RI is the average of five lines. The transcription of each gene in S9 WT corollas is set as 1. Error bar, mean ± SD. Asterisks indicate significant differences from the data in control plants ($^{*}P < 0.05$, $^{**}P < 0.005$, Student's t test).

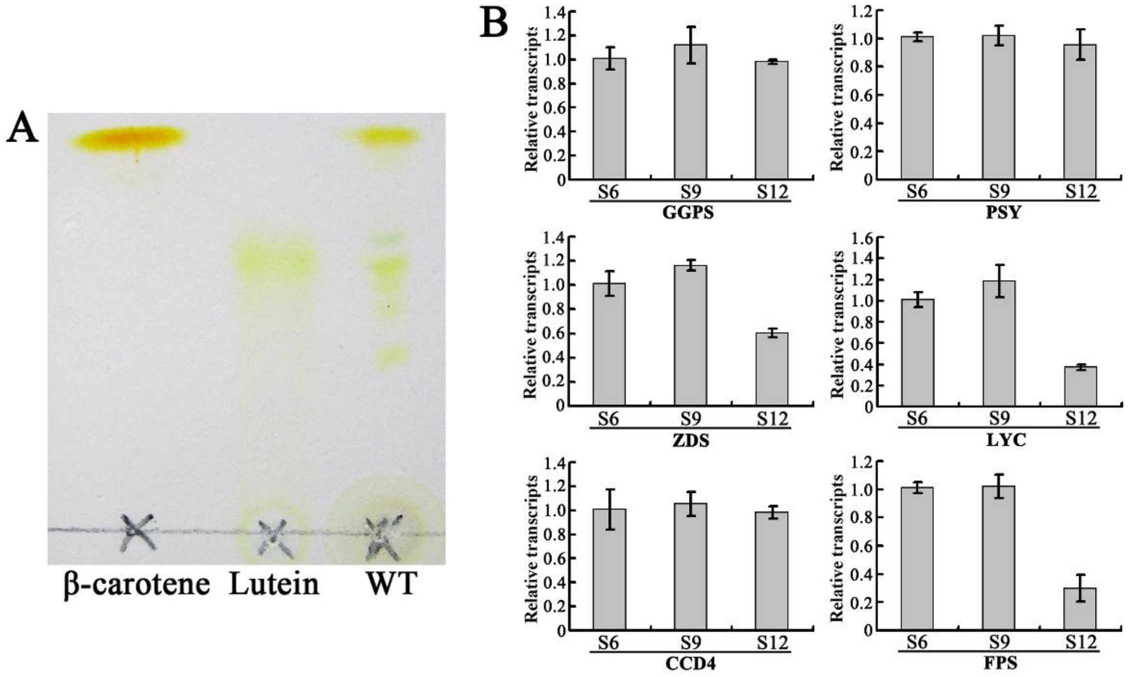

Fig. S5. Carotenoid assay and transcripts of carotenoied synthetic genes in floral nectary.

(A) TLC separation of β-carotene standard, lutein standard and, pigment extract from wild type (WT) nectary. (B) The relative transcript levels of carotenoid synthetic genes in WT nectaries at indicated floral development stages. Gene names and NCBI accessions: *FPS* (farnesyl pyrophosphate synthase; GQ410573.1), *GGPS* (geranylgeranyl diphosphate synthase; GQ911584.1), *PSY* (phytoene synthase; JF461341), *CCD4* (carotenoid cleavage dioxygenase; JF947192.1), *ZDS* (zeta-carotene desaturase; JF975566.1), *LYC* (lycopene cyclase; X81787.1). The values for gene expression levels were determined based on three replicates. The transcription of each gene in S6 nectaries is set as 1. Error bar, mean ± SD.

```
NlsMYB305     1 tcacaaagtaccacatatcttcactgctatcccatatctcttcctctctctcctttttcttcatctttt
NtMYB305      1 -------gtaccacatatcttcactcctatcccatatctcttcctctctctcctttttcttcatttgtt

NlsMYB305    71 tttttttaaaaaaaagatggataaaaaaccatgcaattctcaagatgttgaagtaaggaaaggaccttgg
NtMYB305     64 tttt-------aaaagatggataaaaaaccatgcaactctcaagatgttgaagtgaggaaaggaccttgg

NlsMYB305   141 actatggaagaggatttaattctcattaactacattgctaatcatggtgaaggtgtttggaattccttag
NtMYB305    127 actatggaagaggatttaattctcattaactacattgctaatcatggtgaaggtgtttggaattccttag

NlsMYB305   211 ctaaatctgctggtctcaaacgtaccggaaaaagctgtcggctccggtggctaaattatctccggcctga
NtMYB305    197 ctaaatctgctggtctcaaacgtaccggaaaaagctgtcggctccggtggctaaattatctccggcctga

NlsMYB305   281 tgtccggaggggaaatattacacctgaagaacaactttttgataatggaactgcatgctaagtggggaaac
NtMYB305    267 tgtccggaggggaaatattacacctgaagaacaactttttgataatggaactgcatgctaagtggggaaac

NlsMYB305   351 aggtggtcaaaaattgcaaagcatttgccaggaagaacagataacgagataaagaactattggaggacaa
NtMYB305    337 aggtggtcaaaaattgcaaagcatttgcctggaagaacagataatgagataaagaactattggaggacaa

NlsMYB305   421 ggattcagaagcacataaagcaagcagaaaacatgaatggacaagcagcaaattcagagcaaaatgatca
NtMYB305    407 ggattcagaagcacataaagcaagcagaaaacatgaatggacaagcagctaattcagagcaaaatgatca

NlsMYB305   491 tcaagaaggaagcagtagccatatgtcgtctgctggtccaacagagacttactctccaacttcatactct
NtMYB305    477 tcaagaaggaagcagtagccatatgtcgtctgctggtccagcagagacttactctccaagttcatactct

NlsMYB305   561 gcaaatattgacactacttttcaaggaccttttctcactgaaacaaatgacaacatttggagcatggagg
NtMYB305    547 gcaaatattgacactacttttcaaggcccttttctcactgaaacaaatgacaacatttggagcatggagg

NlsMYB305   631 atatctggtccatgcaattgcttaacggcgattaagtatgttgtttaatattttaatttgagttaaacc
NtMYB305    617 atatctggtccatgcaattgcttaacggcgattaagtatgttgttgaatattttt-atttgagttaaacc
```

Fig. S6. Alignment of ornamental tobacco *MYB305* (*NlsMYB305*) with its common tobacco ortholog *NtMYB305* (KC792284). The alignment was performed using Clone Manager software package (Ver. 8.04).

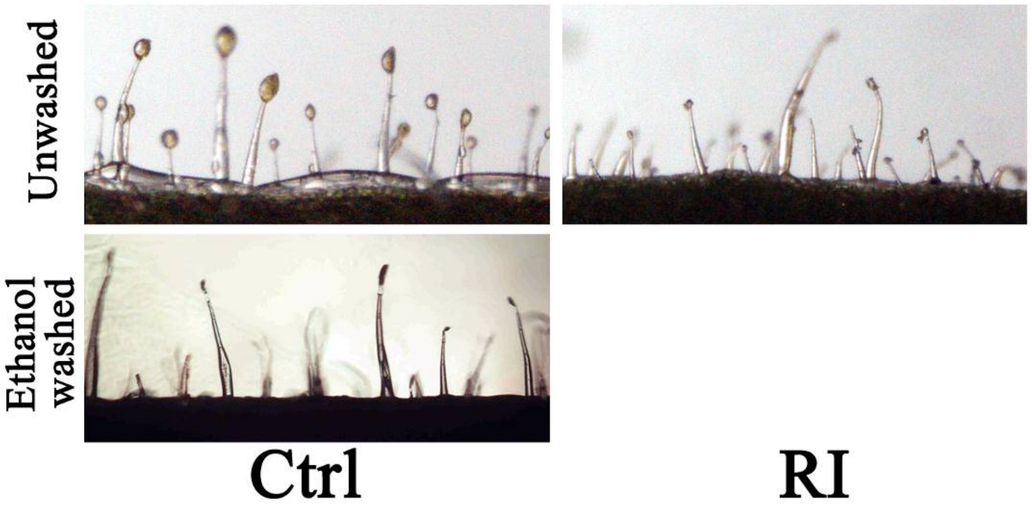

Fig. S7. Morphology of unstained trichomes. Unwashed indicates trichomes directly photographed under a transmitted light microscope. Ctrl, control; RI, *NtCOI1*-silenced. Ethanol washed indicates the Ctrl trichomes photographed after a 5-min wash using 95% ethanol.

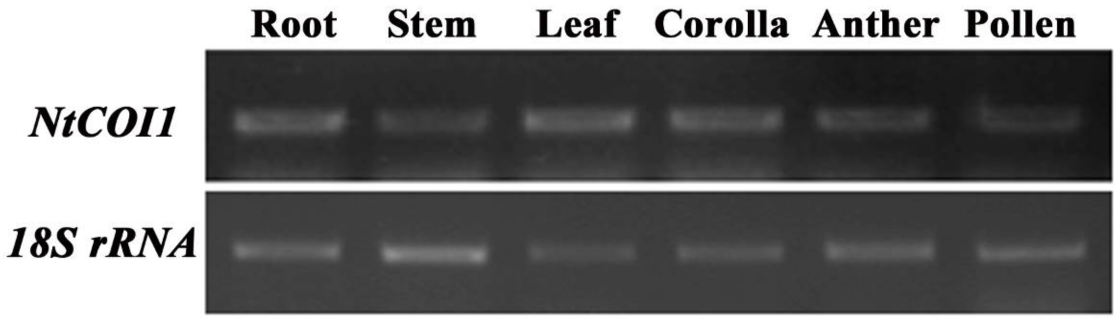

Fig. S8. Spatial expression patterns of *NtCOI1*. *NtCOI1* and *18S rRNA* (as an internal control) genes in indicated tobacco tissues were amplified for 25 cycles and 15 cycles respectively using primers same as those for qRT-PCR. The PCR products were separated on a 1.5% agarose gel with ethidium bromide, and visualized under UV light.

Supplementary Tables:

Table S1. Screening of hygromycin resistant progenies from *NtCOI1*-silenced tobacco produced by pollination with wild type pollen grains.

Transgenic line	RI1	RI2	RI3	RI4	RI5
Number of hygromycin resistant seedlings	44	53	37	55	38
Number of screened seeds	89	91	84	112	74

Table. S2. Oligonucleotide primers used for qRT-PCR.

Gene	Forward Primer	Reverse Primer
NtCOI1	5’-ACAGCAGCCCATTGTTTCTT-3’	5’-GGACATAGAGGGCAAACCTT-3’
4CL	5’-GAAGACGCTGTGAGAACCAA-3’	5’-TCAGTGATGGCAGATCCATT-3’
PAL	5’-TGGTAGGCCTAATTCCAAGG-3’	5’-GAAGTTGCCACCATGTAACG-3’
F3H	5’-GACGAGGCTAGCGAGAGAGT-3’	5’-GGTGGTCCGCATTCTTAAAT-3’
DRF	5’-ATTCCTCACGCCTACATTCC-3’	5’-CTGCTGTGCTTTGGGTAGAA-3’
FPS	5’-ATACACGTCGAGGTCAACCA-3’	5’-CCAACCAAGAGCACTTGAAA-3’
GGPS	5’-AAAGAGTGGTTCAAGCCGTT-3’	5’-TGGCAGCATCAAAGTAGCTC-3’
PSY	5’-TGCTTTGTTGTGGGTTGTTT-3’	5’-ACTCAACAAGCCCAAATTCC-3’
CCD4	5’-TTATTACCCTCGGCCGTTAC-3’	5’-TCACCATCCCACTCTTCAAA-3’
ZDS	5’-AACATTATCGAGGGCCAAAG-3’	5’-AAGGGCAAGAGCTACAGCAT-3’
LYC	5’-GTTCCTCCACTTCTGCCAAT-3’	5’-GAATTTGAGGCCATGGATTT-3’
AGPs	5’-GCTTTCTACAATGCCAATTTGG-3’	5’-ACTGTCTGTGACATCGGCAT-3’
SS2	5’-GGCATACTGCTTTATTGCCA-3’	5’-TGGTGGAAGATCTACGTATGAAA-3’
BAM1	5’-AGCGTGTCAAGCACTAATGG-3’	5’-CTGTCCAACGGCATCATAAC-3’
Nec1	5’-GATGAGGACATGCTCCAAGA-3’	5’-CACCACTGTTTCATGCACAA-3’
INV1	5’-CATCAAGGGTTTATCCGACA-3’	5’-TTGCAACTAGAGCAGTTCGG-3’
INV2	5’-CGGATATCAGCATCACCAAG-3’	5’-GTAGTACTCAAACGCGGCAA-3’
INV3	5’-AATGGATCAAGCCCGATAAC-3’	5’-GGACATTCCCAATTTCCTGT-3’
INV4	5’-GAGTGCTCATTGGAAGCAAA-3’	5’-AGTCGTTCATGAAACCCACA-3’
INV5	5’-ATGCACAATCCGGAAACATA-3’	5’-CCCAAGCAGTAGTTGGGTCT-3’
NtMYB305	5’-CATGGTGAAGGTGTTTGGAA-3’	5’-CATCAGGCCGGAGATAATTT-3’
18S	5’-GAAAGACGAACAACTGCGAA-3’	5’-GAAGGGATACCTCCGCATAG-3’

Supplementary Methods:

Anthocyanin quantification

Anthocyanin was quantified as described by Deikman and Hammer (1995). Pre-weighed fresh tobacco corollas were boiled in 2 ml of extraction buffer (18% 1-propanol, 1% HCl, and 81% water) for 3 minutes, and then incubated in darkness overnight at room temperature.

The absorbances (A_{535} and A_{650}) of the extracts were spectrophotometrically measured. The anthocyanin content was expressed as ($A_{535} - A_{650}$) g^{-1} fresh weight (FW). Three replicates were measured for each plant line.

Supplementary References:

Deikman J, Hammer PE. 1995. Induction of anthocyanin accumulation by cytokinins in Arabidopsis thaliana. *Plant Physiol* **108**, 47-57.
